# Seismic Imaging of the North American Midcontinent Rift Using *S*‐to‐*P* Receiver Functions

**DOI:** 10.1029/2018JB015771

**Published:** 2018-09-19

**Authors:** Ben Chichester, Catherine Rychert, Nicholas Harmon, Suzan van der Lee, Andrew Frederiksen, Hao Zhang

**Affiliations:** ^1^ National Oceanography Centre Southampton, Ocean and Earth Sciences University of Southampton Southampton UK; ^2^ Department of Earth and Planetary Sciences Northwestern University Evanston IL USA; ^3^ Department of Geological Sciences University of Manitoba Winnipeg Manitoba Canada; ^4^ Department of Geology and Geophysics University of Utah Salt Lake City UT USA

**Keywords:** Midcontinent Rift, receiver functions, crustal underplating, MLD, rifting, SPREE

## Abstract

North America's ~1.1‐Ga failed Midcontinent Rift (MCR) is a striking feature of gravity and magnetic anomaly maps across the continent. However, how the rift affected the underlying lithosphere is not well understood. With data from the Superior Province Rifting Earthscope Experiment and the USArray Transportable Array, we constrain three‐dimensional seismic velocity discontinuity structure in the lithosphere beneath the southwestward arm of the MCR using *S*‐to‐*P* receiver functions. We image a velocity increase with depth associated with the Moho at depths of 33–40 ± 4 km, generally deepening toward the east. The Moho amplitude decreases beneath the rift axis in Minnesota and Wisconsin, where the velocity gradient is more gradual, possibly due to crustal underplating. We see hints of a deeper velocity increase at 61 ± 4‐km depth that may be the base of underplating. Beneath the rift axis further south in Iowa, we image two distinct positive phases at 34–39 ± 4 and 62–65 ± 4 km likely related to an altered Moho and an underplated layer. We image velocity decreases with depth at depths of 90–190 ± 7 km in some locations that do not geographically correlate with the rift. These include a discontinuity at depths of 90–120 ± 7 km with a northerly dip in the south that abruptly deepens to 150–190 ± 7 km across the Spirit Lake Tectonic Zone provincial suture. The negative phases may represent a patchy, frozen‐in midlithosphere discontinuity feature that likely predates the MCR and/or be related to lithospheric thickness.

## Key Points

- *S*‐to‐*P* receiver functions constrain a Moho, crustal underplating and midlithosphere discontinuities beneath the Midcontinent Rift
- The Moho and crustal underplating directly beneath the rift axis vary in character from north to south
- Midlithospheric discontinuities predate the rifting event and seem unperturbed by rift‐related activity

## 1 Introduction

The shallow, dense igneous rocks of North America's 1.1‐Ga failed Midcontinent Rift (MCR) System create a striking magnetic anomaly and gravity high that demarcate the rift axis (Chase & Gilmer, 1973; Hinze et al., 1992; King & Zietz, 1971). The system consists of two arms that converge in the Lake Superior region—one extending southeast through Michigan and one extending southwest through Minnesota, Wisconsin, and Iowa to Kansas—overall spanning a distance of ~3,000 km (Hinze et al., 1992). Despite its size and prominence in geophysical surveys, the formation, evolution, and failure are still being debated and particularly at a lithospheric scale are not well understood.

A leading question for the MCR is whether rifting was initiated via passive or active rifting. Passive rifting attributes rift initiation to lithospheric tensional stresses that cause extension and thinning of the lithosphere, generating passive decompression upwelling of mantle material. Active rifting attributes impingement of anomalous large‐scale hot mantle upwelling and magmatism as the cause, driving thermal erosion and lithospheric weakening that leads to isostatic uplift, which causes tensional stresses (White & McKenzie, 1989). Many geochemical and isotopic studies of exposed igneous rocks related to the MCR point toward early melting of an enriched plume source (Davis & Green, 1997; Nicholson et al., 1997; Vervoort et al., 2007; White, 1997), indicating an active rift environment for the MCR. On the contrary, based upon temporal and spatial proximity of the MCR and the Grenville Province, early studies attribute the cause of rifting to Grenville collisional events during the 1.3–0.98‐Ga Grenville Orogeny (Gordon & Hempton, 1986). More recently, it was proposed that the rifting event instead occurred during a lapse in local compression related to the Grenville Orogeny and ceased when motion was taken up by seafloor spreading between the supercontinents Amazonia and Laurentia (Stein et al., 2014).

This paper aims to further constrain velocity discontinuities beneath the 1.1‐Ga MCR, in order to shed light on its initiation mechanism and the events that caused it to cease. Data used include earthquake arrivals detected by the Superior Province Rifting Earthscope Experiment (SPREE; Wolin et al., 2015), the Earthscope Transportable Array, and the US backbone network on the rift and surrounding area. Here we use *S*‐to‐*P* (*Sp*) receiver functions to illuminate the depth and character of seismic velocity discontinuities throughout the lithosphere and how they vary laterally over the rift and its flanks along the southwestward arm. Constraining variations in the crust‐mantle boundary and other lithospheric features aids the evaluation of the extent and role of magmatism during the 1.1‐Ga rifting event, in that evidence of lithospheric alteration from past events of magmatism may still remain. Finding the presence or not of these signatures and constraining them at a fine scale are important for a better understanding of rifting dynamics and how a rift may initiate and subsequently cease in the confines of a continent.

## 2 Background

Along the arms of the MCR, away from the Lake Superior region, volcanic rocks associated with the MCR rifting event are mostly buried by younger sedimentary deposits (Allen et al., 2006). There are outcrops and drill samples collected that reveal dense mafic rocks overlain by less dense sedimentary sequences, although these samples are few (Ojakangas et al., 2001). Thus, in order to study the nature of the rift along the rift arms, studies must rely on interpretation of seismological, gravitational, and magnetic data and extrapolation from studies of sparse outcrops. Seismic profiling of Lake Superior and the rift's arms and extensive geochemical and isotopic studies of exposed and well‐preserved Lake Superior rocks have already constrained a complex history of the MCR rifting events, including crustal extension (Green et al., 1989; Hinze et al., 1992), volcanism that filled the rift basin with large volumes of flood basalts (Paces & Miller, 1993; Vervoort et al., 2007), and thermal subsidence and sedimentation (Cannon, 1992). Seismic profiles also reveal thrust faults that bound the rift axis in Lake Superior and the southwestern arm, which are inverted normal faults that primarily accommodated extension (Chandler et al., 1989; Hinze et al., 1992). Raising of the central graben of the rift along these reverse faults is thought to have begun ~10–20 Myr after extension had ceased and is a result of compression in the 1.3–0.98‐Ga Grenville Orogeny (Cannon, 1994). Crustal shortening lasted ~20 Myr and amounted to a shortening of ~30 km in the southwestern arm of the rift, based on seismic reflection profiling that recognizes marker horizons between flood‐basalt sequences and overlying sedimentary rocks (Cannon, 1994).

The prerift crust is likely to have undergone extensional crustal thinning to less than one third of its original thickness (Cannon et al., 1989; Hutchinson et al., 1990)—however, given its complex history, the resulting MCR crust and mantle structure cannot be simply characterized by rift‐related thinning. Instead, seismic imaging suggests the existence of multiple layers of crust and upper mantle within the MCR. Beneath the Lake Superior portion of the rift, active‐source reflection seismic surveys reveal shallow crustal additions up to 30 km thick, interpreted to consist of extruded flood basalts during rifting and mainly clastic sediments deposited in the thermally subsiding rift basin (Hinze et al., 1992). Furthermore, underplating of the crust has been inferred by the presence of intermediate density and seismic velocity between the crust and mantle beneath the rift axis in Lake Superior based on gravity modeling and reflection seismic images (Hutchinson et al., 1990). Receiver function studies have also observed weaker or incoherent phases that are suggestive of a more complex transition from rift crust to mantle beneath the southwestern arm of the MCR (Moidaki et al., 2013; Shen et al., 2013; Zhang et al., 2016), where three‐dimensional density modeling also requires intermediate density at the base of the rift crust to satisfy the gravity and topography variations (Levandowski et al., 2015). *P*‐to‐*S* (*Ps*) waveform fitting and *H‐κ* stacking reveal a crust‐mantle transitional layer that is inferred as crustal underplating, up to 25 km thick, beneath SPREE stations located over the rift axis beneath the northern segment (Wisconsin‐Minnesota border) and middle segment (Minnesota) of the rift, which is bounded by two weaker discontinuities, the lower of which extends to depths of up to 60 km (Zhang et al., 2016). Further south beneath the southern segment of the rift (Iowa), using a more sparsely distributed, across‐rift station line, *H‐κ* stacking has constrained phases related to a velocity increase with depth that are weaker at two stations on the rift axis than the rift flanks and that occur at depths of up to 53 km (Moidaki et al., 2013).

## 3 Method

### 3.1 Data

**Figure 1:**
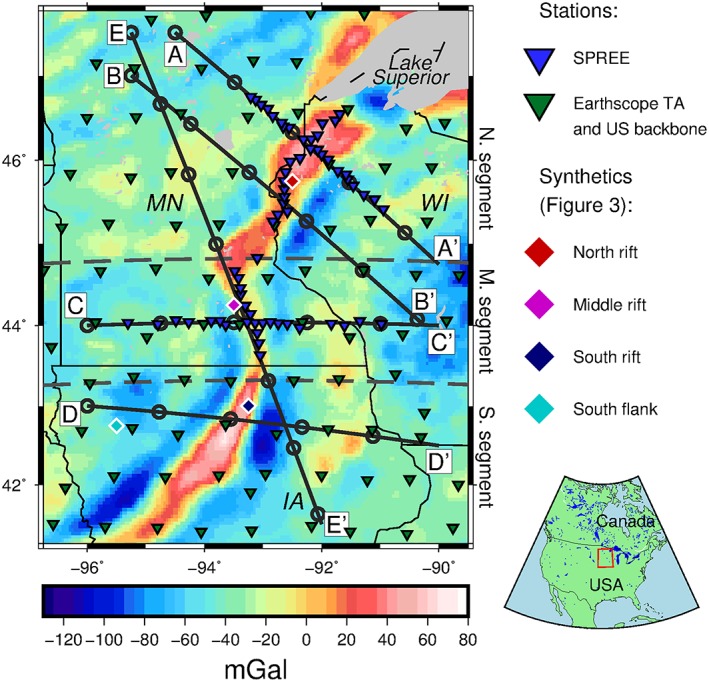
Bouguer gravity anomaly map of the study region—the western arm of the Midcontinent Rift. Inverted triangles show the location of the seismic stations: blue, Superior Province Rifting Earthscope Experiment (SPREE); green, Earthscope Transportable Array (TA) and US backbone. Diamonds show locations of synthetics in Figure 3. Cross sections, A–E, across the rift presented in Figures 5 and 6 are shown. Gray dashed lines at 44.75°N and 43.25°N separate the northern, middle, and southern segments that are used for descriptive purposes in text. Thin black lines indicate state boundaries. States are labeled: MN, Minnesota; WI, Wisconsin; IA, Iowa. Gray area in the northeast is Lake Superior. Bouguer gravity anomaly data used from Kucks (1999). Red box in inset (bottom right) shows study region.

Two data sets are used: teleseismic earthquakes recorded from January 2011 to December 2013 by 82 seismic broadband stations from SPREE (Wolin et al., 2015); and teleseismic earthquakes recorded from 1996 to 2014 by the US backbone station network and the Earthscope Transportable Array stations deployed at roughly 70‐km spacing in the region, both together we refer to as the backbone array. The SPREE stations were mainly located over the rift axis, with 66 of the stations distributed along the rift axis and in two rift‐perpendicular lines (Figure 1)—the other 16 stations are located in Ontario north of Lake Superior. Events located at epicentral distances of 55–80° from the stations are used (Rychert et al., 2007; Wilson et al., 2006). Events with *M*_*w*_ *≥* 5.0 are considered from SPREE, and events with *M*_*w*_ *≥* 5.8 are considered from the backbone array, forming a data set of 25,401 event‐station pairs: 15,901 event‐station pairs from the backbone array and 9,500 from SPREE.

### 3.2 *S*‐to‐*P* Receiver Functions With Extended‐Time Multitaper Deconvolution

Each event is rotated into theoretical *P* and *S* components using a free‐surface transformation matrix (Bostock, 1998). We divided the region into areas that are thickly sedimented (approximately *≥*1 km thick) and those that are not based on waveform fitting of *Ps* receiver functions beneath SPREE stations by Zhang et al. (2016). In locations with thick sediment, we assumed surface velocities *V*_*p*_ = 4.00 km/s and *V*_*s*_ = 2.00 km/s; otherwise, *V*_*p*_ = 5.90 km/s and *V*_*s*_ = 3.41 km/s. The parent *S* wave is manually picked, thereby eliminating any unclear *S* wave arrivals. After this elimination, we were left with 7,964 event‐station pairs in the study region (5,582 backbone array and 2,382 SPREE). Each parent *S* wave is deconvolved from the daughter signal using extended‐time multitaper deconvolution (Helffrich, 2006; Rychert et al., 2012) to calculate the receiver functions. Receiver functions that from 0 to 70 s exhibit same‐frequency, similar amplitude signals were deemed unstable and are eliminated by inspection, leaving 5,279 *Sp* receiver functions (3,162 backbone array and 2,217 SPREE). We multiply the receiver functions by −1 so that a positive amplitude indicates a velocity increase with depth and a negative amplitude indicates a velocity decrease with depth, consistent with *Ps* receiver function studies.

**Figure 2:**
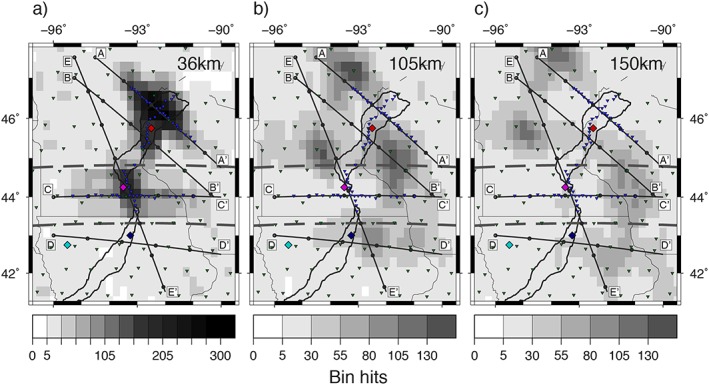
Receiver function hit count maps of each bin at depths (a) 36, (b) 105, and (c) 150 km. Inverted triangles show the location of the seismic stations: blue, Superior Province Rifting Earthscope Experiment (SPREE); green, Earthscope Transportable Array and US backbone. Diamonds show locations of synthetics in Figure 3. Cross sections, A–E, across the rift presented in Figures 5 and 6 are shown. Gray dashed lines at 44.75°N and 43.25°N separate the northern, middle, and southern segments that are used for descriptive purposes in text. Solid black lines describe the rift axis (Kucks, 1999). Thinner black lines indicate state boundaries.

A band‐pass filter of 0.02 to 0.5 Hz is applied to the deconvolved waveforms, which are then migrated to depth along the ray paths of the respective *Sp* phase and stacked into a 0.25° *×* 0.25° *×* 1‐km grid (Angus et al., 2009; Rychert et al., 2012). Grid points (bins) with less than five hits are discarded, and the grids are subsequently smoothed according to the Fresnel zone (Fowler, 2005) of each waveform, with a minimum width of 20 km. Hit counts in each bin at depths of 36, 105, and 150 km are presented in Figure 2.

### 3.3 Migration Model

The one‐dimensional migration model for each receiver function is obtained by tracing the approximate *Sp* ray path through a three‐dimensional Earth model that is based upon previously determined rift and near‐rift Earth structure (Zhang et al., 2016) and US‐CrustVs‐2015 Moho depths (Schmandt et al., 2015) in the outer bounds of the grid. For the rifted region, Moho beneath the flanks, the base of an underplate layer beneath the rift axis, and the crustal *V*_*p*_/*V*_*s*_ values are defined, which we base on *H‐κ* stacking and waveform fitting results beneath the SPREE stations (Zhang et al., 2016). We assumed these values extend both further north and also south along the rift axis to fill in the larger area considered in our study. The grid then undergoes interpolation to create a grid with 5‐km spacing and Gaussian smoothing over a 10‐km length scale. The Earth model also includes a sediment layer over the rift. The sediment extent is based on the extent of the Bouguer anomaly map (Kucks, 1999) that illuminates the rift—the high Bouguer anomaly is assumed to be the *rift*—and sediment depth is based on Zhang et al. (2016). The final crustal thickness used for migration is determined by the Moho piercing point of each waveform in the above model. The crustal *P* wave velocity structure is designed to be a linear gradient centered on the average *P* wave velocity of the North American crust (Zhang et al., 2016): *V*_*p*_ = 5.90 km/s at surface and *V*_*p*_ = 6.90 km/s at the crustal thickness of the Earth model described above. In areas of sediment, we assume a linear gradient through the sediment and the crust with *V*_*p*_ = 4.00 km/s at the surface, *V*_*p*_ = 5.90 km/s at the base of the sediment, and the same linear gradient to *V*_*p*_ = 6.90 km/s at the crustal thickness in the Earth model. For the mantle, we assume values from IASP91 (Kennet, 1991).

### 3.4 Error

Uncertainties for discontinuity depths are defined by changing the *V*_*p*_/*V*_*s*_ ratio in the model used for migration by 0.1 via changes to *V*_*s*_, which encompasses extreme mantle lithosphere values predicted by compositional variations (Hacker & Abers, 2004) and most crustal variations. Increasing/decreasing the *V*_*p*_/*V*_*s*_ ratio by 0.1 results in midlithospheric discontinuities that are 7 km shallower/deeper on average. The Moho phase is 4 km shallower/deeper on average when this change is applied.

Uncertainties for the amplitudes of the observed phases are defined by the 95% confidence limits, assuming Gaussian statistics, based on the standard error of the mean of the stacked receiver function amplitudes in the associated bins.

### 3.5 Synthetic Waveform Modeling

**Figure 3:**
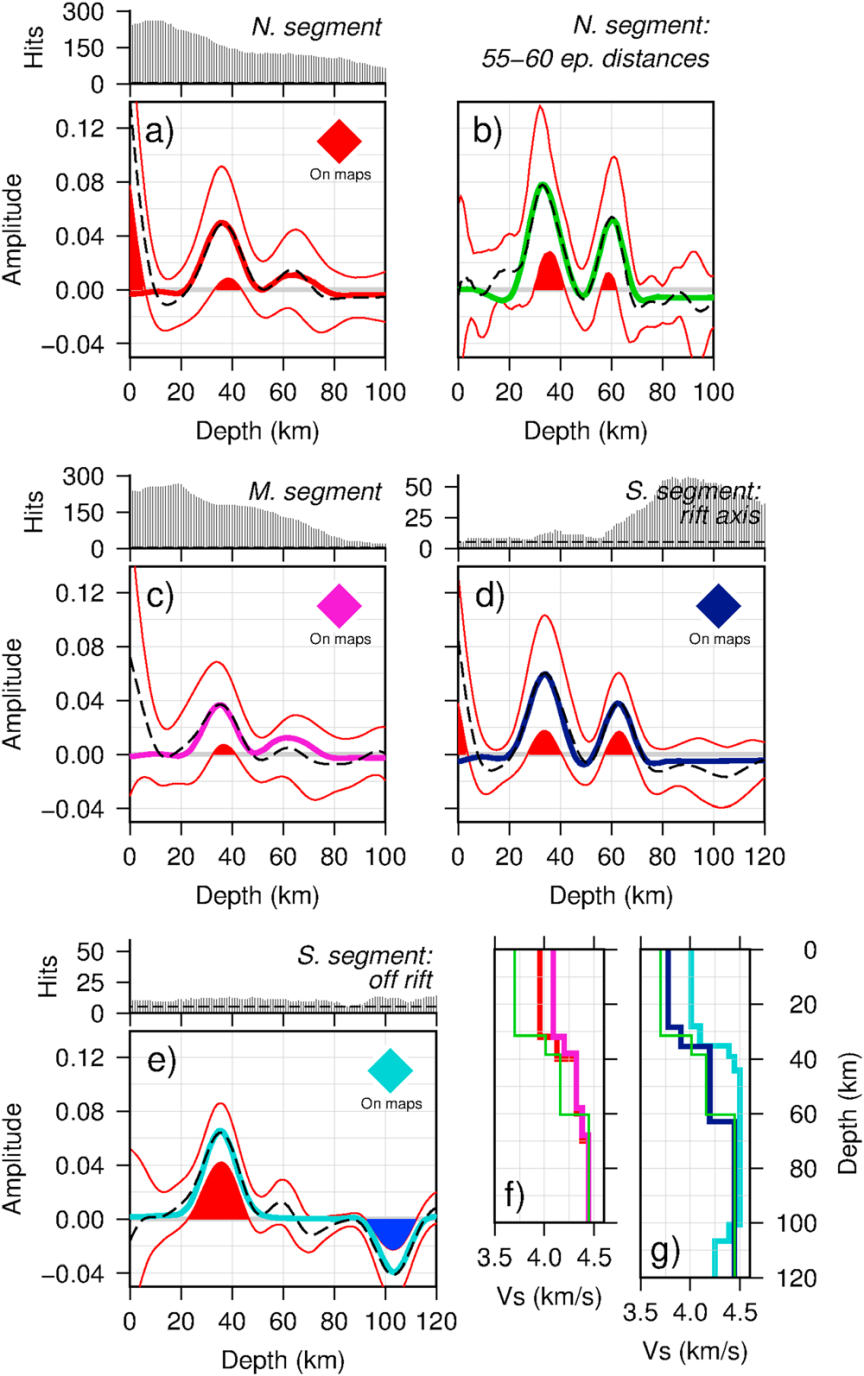
Synthetic waveform modeling of stacked *S*‐to‐*P (Sp)* receiver functions. Colored solid lines, synthetic waveforms; black dashed lines, our *Sp* data; thin red lines, 95% confidence limits; shaded red (positive phase) and blue (negative phase) regions indicate where data are significant from zero. Above plots, hit count of receiver functions in each 1‐km‐depth bin of the smoothed grid at location. Dashed lines indicate five hits (the minimum accepted). (a) *Northern segment:* Stacked receiver functions at −92.5°, 45.75°, from the smoothed grid. (b) *Northern segment:* Stacked receiver functions using only waveforms from 55° to 60° epicentral distances. (c) *Middle segment:* Stacked receiver functions at −93.5°, 44.25°, from the smoothed grid. (d) *Southern segment:* Stacked receiver functions at −93.25°, 43°, from the smoothed grid. (e) *Southern segment:* Stacked receiver functions at −95.5°, 42.75°, from the smoothed grid. (f/g) Shear‐wave velocity‐depth profiles producing each synthetic waveform, which are color coded to the synthetic waveforms in (a–e). The velocity‐depth profile for the 55–60° northern segment waveform (b, green) is shown in both (f) and (g) for comparison. Although we present the result in terms of an absolute shear velocity as an example, our only constraint is on the magnitude of relative velocity contrasts in depth.

We perform synthetic waveform modeling of discontinuity structures representative of different locations in the study region. For each location, we forward model a shear velocity‐depth profile for a one‐dimensional slice of our gridded and stacked receiver functions using one‐dimensional reflectivity synthetic waveforms (Shearer & Orcutt, 1987). Only phases that are significant from zero according to 95% confidence limits are modeled. Processing of the synthetic waveforms is equivalent or similar to that used in producing the *Sp* receiver functions—a band‐pass filter of 0.02 to 0.5 Hz is applied, and the migration model used is based on the location of each synthetic waveform. We use a ray parameter of 0.1058 s/km, which is the average of the values used in the study using the entire epicentral distance range of 55–80°. In a test to constrain complex structure beneath the rift using a smaller epicentral distance range of 55–60° (section 4.3), a ray parameter of 0.1178 s/km is used. In the synthetic models, *V*_*p*_/*V*_*s*_ ratios of 1.72 and 1.80 are used to define the velocity‐depth profiles for the crust and mantle, respectively. We use an upper crustal density of 2.80 g/cm^3^, a lower crustal density of 2.95 g/cm^3^, and a mantle density of 3.32 g/cm^3^. The synthetic waveforms and required shear‐wave velocity‐depth profiles are presented in Figure 3.

## 4 Results

### 4.1 Positive Velocity Discontinuities

**Figure 4:**
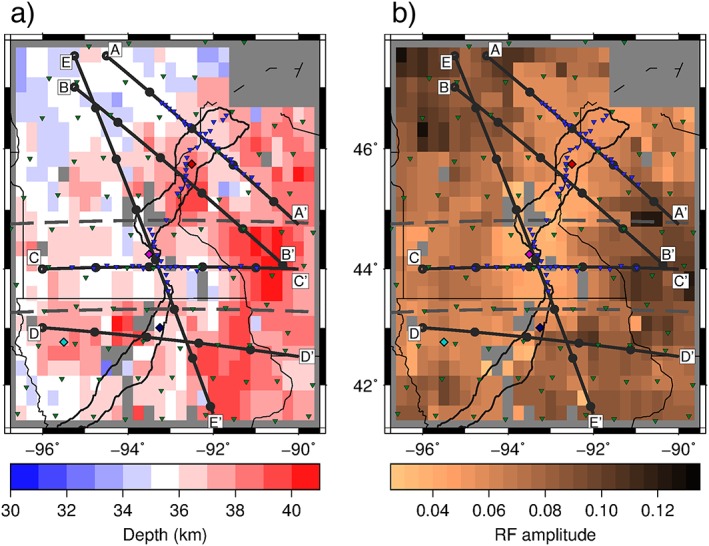
Moho depths and corresponding *S*‐to‐*P* receiver function amplitude. (a) Depth to the seismic velocity increase associated with the Moho. (b) Corresponding amplitude of the receiver function phase at depths on the left in (a). Positive amplitudes correspond to a velocity increase with depth. Each block corresponds to a 0.25° × 0.25° bin that into which receiver functions are stacked. Gray boxes within the study region represent locations that are not significant from zero based on 95% confidence limits. Inverted triangles show the location of the seismic stations: blue, Superior Province Rifting Earthscope Experiment (SPREE); green, Earthscope Transportable Array and US backbone. Diamonds show locations of synthetics in Figure 3. Cross sections, A–E, across the rift presented in Figures 5 and 6 are shown. Gray dashed lines at 44.75°N and 43.25°N separate the northern, middle, and southern segments that are used for descriptive purposes in text. Solid black lines describe the rift axis (Kucks, 1999). Thinner black lines indicate state boundaries.

The *Sp* receiver functions illuminate a positive polarity phase (seismic velocity increase with depth) that persists over the study region at depths in the range of 33–40 ± 4 km (Figure 4), consistent with Moho depths in the region (Zhang et al., 2016). Little depth variation from rift flank to rift axis exists outside of our 4‐km error bounds. At a distance of ~200–300 km east of the rift axis, there is possibly slightly thicker crust throughout most of the region with Moho depths of 37–40 ± 4 km. There appears to be a strong spatial correlation between the rift axis and a weak Moho phase (amplitudes less than 0.06). Away from the rift axis, amplitudes are stronger with values of 0.07–0.12 ± 0.04 in the east and northwest of the study region and values of 0.06–0.08 ± 0.04 in the west and south.

**Figure 5:**
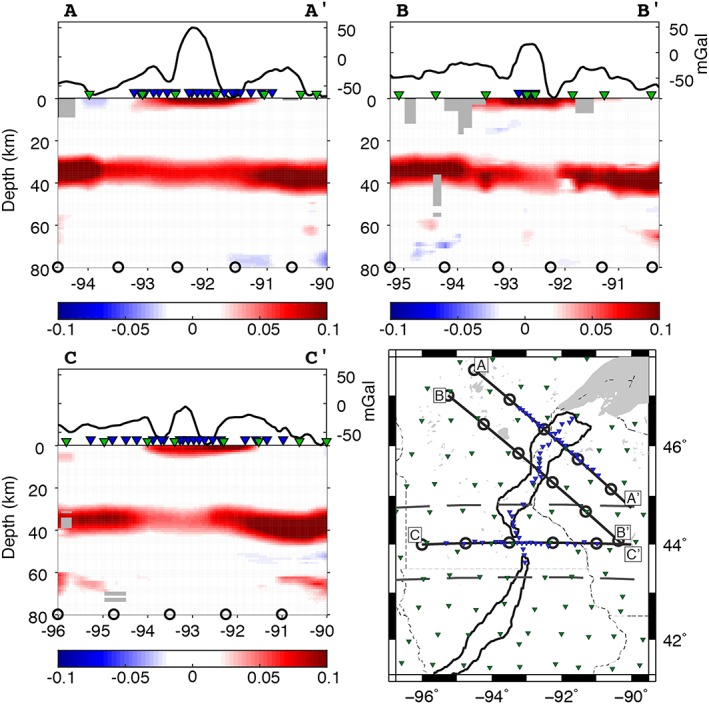
Cross sections A–C through the Midcontinent Rift, showing the migrated *S*‐to‐*P* receiver function phases at depth. *A–C:* Cross sections corresponding to lines depicted in the reference map (bottom right) and in Figures 1, 2, and 4. The colors indicate the polarity of the seismic discontinuities from receiver functions: red, positive polarity (seismic velocity increase with depth); blue, negative polarity (seismic velocity decrease with depth). Amplitude color bars are shown below each cross section. Circles plotted at depth have a 100‐km lateral spacing and correspond to circles along the lines in the reference map and Figures 1, 2, and 4. Gray boxes signify bins that have less than five hits. Bouguer anomaly is plotted above each cross section in units of mGal (Kucks, 1999), where proximal stations are also indicated as inverted triangles—blue, Superior Province Rifting Earthscope Experiment (SPREE); green, Earthscope Transportable Array and US backbone. *Bottom right:* reference map. Solid black lines describe the rift axis (Kucks, 1999). Thinner black lines indicate state boundaries. Gray area in the northeast is Lake Superior. Gray dashed lines at 44.75°N and 43.25°N separate the northern, middle, and southern segments. Inverted triangles show the location of the seismic stations: blue, SPREE; green, Earthscope Transportable Array and US backbone.

In the northern segment of the rift (north of 44.75°N), the amplitude of the Moho phase beneath the rift is largest, 0.06 ± 0.04, in the north of the segment decreasing to 0.04 ± 0.03 toward the middle segment. Amplitudes of phases lower than 0.04 in the south of the segment are so low that they are typically not significant from zero. The amplitude generally remains as low as 0.07 ± 0.04 up to 100 km from the rift axis on both flanks. Depths of the Moho phase in the northern segment are relatively constant, with values of 34–38 ± 4 km, excluding a small area just offset from the rift in the south of the segment with 39–40 ± 4‐km depth. Cross sections A–A′ and B–B′ (Figure 5) illustrate the variation in depth and amplitude over the rift in the northern segment.

Rift Moho amplitudes in the middle segment (between latitudes 43.25°N and 44.75°N) are the weakest of the study, similar to the southern portion of the northern segment, with values of 0.04–0.05 ± 0.03, or less (Figure 5 cross section C–C′). Similar to the northern segment, amplitudes remain low up to ~100 km from the rift, strengthening away from the rift. Depths are also relatively constant in the middle segment, with values of 34–37 ± 4 km. We observe deeper values of 38–40 ± 4 km toward the east.

**Figure 6:**
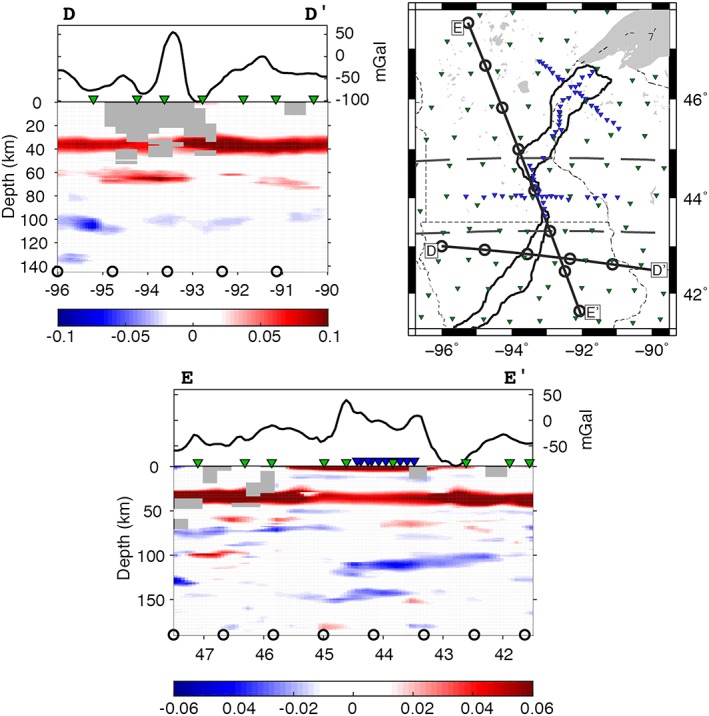
Cross sections D–E through the Midcontinent Rift, showing the migrated *S*‐to‐*P* receiver function phases at depth. *D‐E:* Cross sections corresponding to lines depicted in the reference map (top right) and in Figures 1, 2, 4, and 7. The colors indicate the polarity of the seismic discontinuities from receiver functions: red, positive polarity (seismic velocity increase with depth); blue, negative polarity (seismic velocity decrease with depth). Amplitude color bars are shown below each cross section. Circles plotted at depth have a 100‐km lateral spacing and correspond to circles along the lines in reference map and Figures 1, 2, 4, and 7. Gray boxes signify bins that have less than five hits. Bouguer anomaly is plotted above each cross section in units of mGal (Kucks, 1999), where proximal stations are also indicated as inverted triangles—blue, Superior Province Rifting Earthscope Experiment (SPREE); green, Earthscope Transportable Array and US backbone. *Top right:* reference map. Solid black lines describe the rift axis (Kucks, 1999). Thinner black lines indicate state boundaries. Gray area in the northeast is Lake Superior. Gray dashed lines at 44.75°N and 43.25°N separate the northern, middle, and southern segments. Inverted triangles show the location of the seismic stations: blue, SPREE; green, Earthscope Transportable Array and US backbone.

The low amplitude beneath the rift in the middle segment extends to the northernmost section of the southern segment (south of 43.25°N), that is, 0.04 ± 0.03. Otherwise, in the southern segment, the rift is characterized by Moho amplitudes of 0.05–0.07 ± 0.03. The western part of the segment is characterized by amplitudes of 0.06–0.08 ± 0.03, and the east of the segment has amplitudes of 0.07–0.11 ± 0.03. Depths of the Moho phase are 34–40 ± 4 km in the southern segment. We illuminate an additional deeper velocity increase beneath the rift axis, which is also offset to the west, at a depth of 62–65 ± 4 km with amplitudes of up to 0.05 ± 0.02 (Figure 6, cross section D–D′).

### 4.2 Negative Velocity Discontinuities

**Figure 7:**
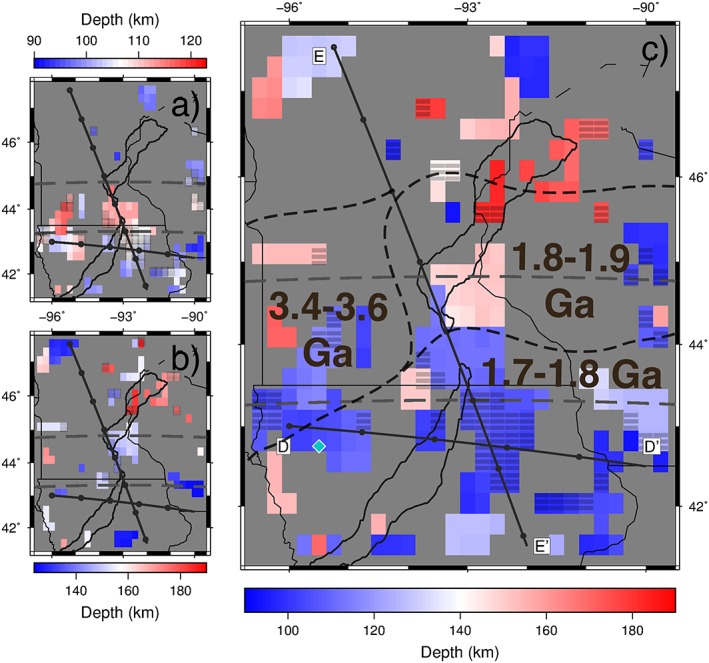
Depths of *S*‐to‐*P* receiver function negative polarity phases (seismic velocity decrease with depth). Note different depth scales for each plot. All phases shown have an amplitude of >0.02. (a) Phases between 90‐ and 123‐km depth. (b) Phases between 124‐ and 190‐km depth. (c) Phases between 90‐ and 190‐km depth—only the largest phase is shown at each grid point. The black dashed lines indicate the boundaries between the Minnesota River Valley Subprovince (age 3.4–3.6 Ga), the Yavapai Province (age 1.7–1.8 Ga), and the Penokean Orogeny (age 1.8–1.9 Ga). Cyan diamond shows location of off‐rift synthetics in Figure 3e. *All panels:* Each block corresponds to a 0.25° × 0.25° bin that receiver functions are stacked in. Barred grid points signify locations where the shown negative phase is not significant from zero according to 95% confidence limits of the amplitude. Cross sections, D and E, across the rift presented in Figure 6 are shown. Gray dashed lines at 44.75°N and 43.25°N separate the northern, middle, and southern segments. Solid black lines describe the rift axis (Kucks, 1999). State borders are marked by thinner black lines.

We image negative polarity receiver function phases (seismic velocity decrease with depth) that are less spatially persistent over the study region than the positive polarity phase we see at Moho depths (Figure 7). Amplitudes of the negative phases across the study are typically 0.03–0.04 ± 0.02. We plot its depth for all bins with five or more waveforms and with barred hatching indicate bins that are not significant from zero.

A bimodal depth distribution of the negative phases is apparent across the study region. Depths of 90–120 ± 7 km predominate the southern segment and extend into the middle segment, and this phase includes a feature that dips to the north from ~100‐km depth to ~115 km. Depths of 150–190 ± 7 km predominate the northern segment and also extend into the middle segment, although the phase at these depths is patchier.

### 4.3 Synthetic Waveforms

In the northern segment, we perform synthetic waveform modeling on two receiver function stacks related to the rift: (1) A one‐dimensional depth profile from our stacked and smoothed grid at a location on the rift axis, which requires a 4% increase in shear velocity with depth at 32.0 km, followed by an increase of 4.5% at 40.0 km (Figure 3a, red). Additionally, we include an increase of 1.5% at 60.0 km and an increase of 1.5% at 70.0 km—these are not required to fit the data and are included to reach a normal mantle shear velocity of 4.44 km/s. (2) In an attempt to resolve a deeper positive phase in the northern and middle segments detected by *Ps* waveform modeling (Zhang et al., 2016), we stack waveforms whose ray paths pierce the deeper structure beneath the rift axis and test for earthquake back‐azimuth and epicentral distance dependencies. We find no dependency on the back‐azimuth. We do observe a double positive phase in the northern segment if we stack receiver functions of this selection from earthquakes located at epicentral distances of 55–60°, possibly due to larger expected conversion transmission coefficients for smaller epicentral distances (Rychert et al., 2007). Synthetic waveform modeling of this stack requires an 8% increase in shear velocity with depth at 31.5 km, followed by an increase of 3.5% at 38.5 km and an increase of 6.5% at 60.5 km (Figure 3b, green).

Synthetic waveform modeling of the rift axis in the middle segment requires a 2.5% increase in shear velocity with depth at 32.0 km, followed by a 3% increase at 38.0 km (Figure 3c, purple). We include an increase of 1.5% at 58.0 km and an increase of 1.5% at 68.0 km—again these are not required to fit the data and are included to reach normal mantle shear velocities.

Synthetic waveform modeling of the rift axis in the southern segment requires a 3.5% increase in shear velocity with depth at 28.5 km, followed by a 7% increase at 35.5 km and a 5.5% increase at 63.0 km (Figure 3d, dark blue). Modeling of the flank requires a 2% increase at 28.2 km, followed by a 6.5% increase at 35.2 km and 2.5% from 39.2 km to 44.2 km. A shear velocity decrease with depth of 5.5% over 6 km centered at 103.7 km is required for the deeper negative phase in this stack (Figure 3e, cyan).

## 5 Discussion

### 5.1 Positive Velocity Discontinuities

**Figure 8:**
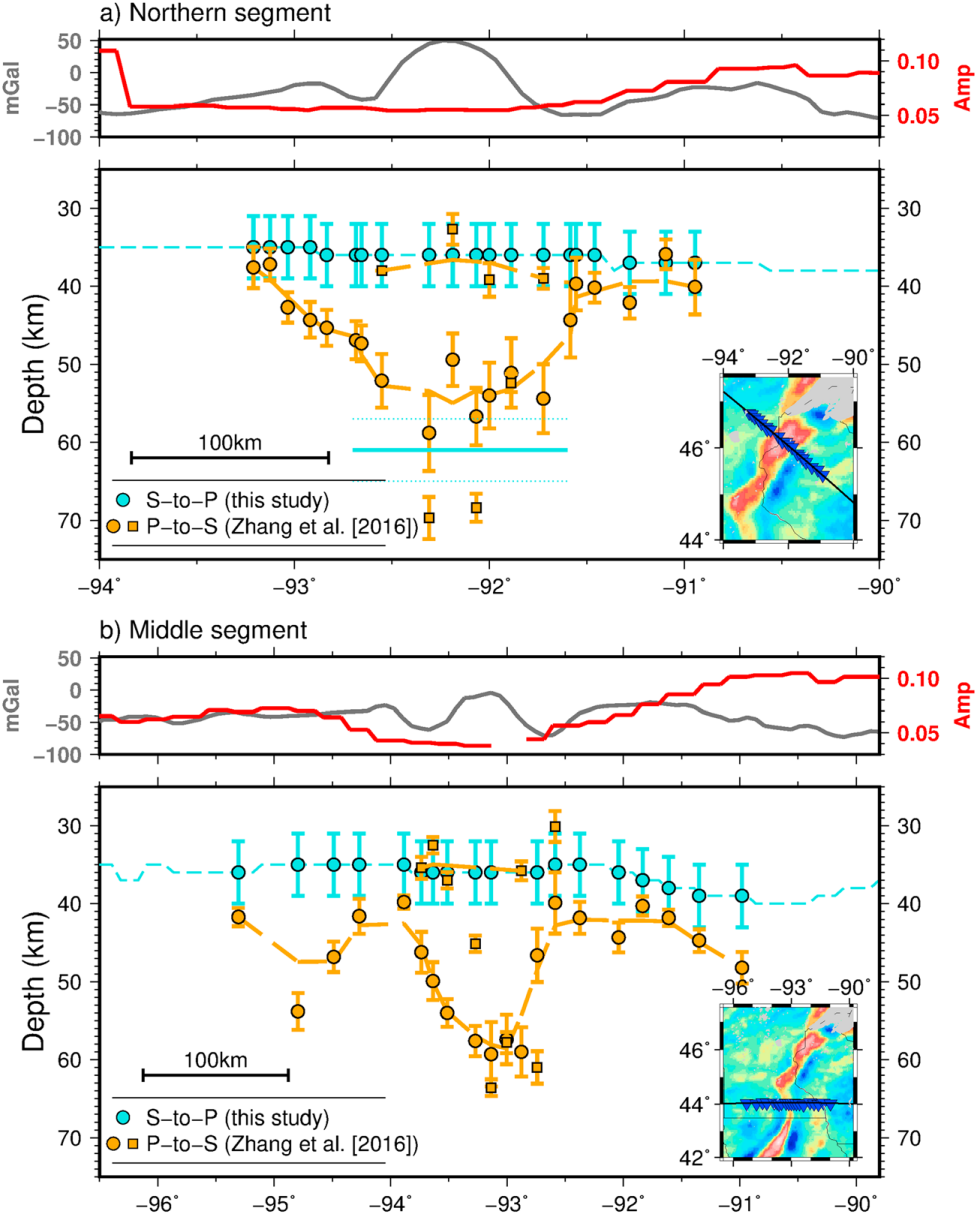
Comparison between *S*‐to‐*P* (*Sp*; this study) and *P*‐to‐*S* (*Ps*; Zhang et al., 2016) positive phases in receiver functions (seismic velocity increase with depth) beneath Superior Province Rifting Earthscope Experiment (SPREE) stations across the MCR. (a) Northern segment and (b) middle segment. Reference Bouguer anomaly maps (Kucks, 1999) in bottom right of each plot—inverted blue triangles are the SPREE stations. Blue, *Sp*: circles are depths beneath each SPREE station; dashed line is depth across the whole cross section; solid line that represents depth that hints of a deeper positive phase are seen using earthquakes from 55° to 60° in the northern segment. Orange, *Ps:* circles are depths illuminated by waveform fitting techniques; dashed line is moving average of those; squares are depths illuminated by *H‐κ* stacking techniques. Above each plot Bouguer gravity anomaly along the cross section (gray) and the amplitude of *Sp* positive phase (red). Gaps in lines are where the associated receiver functions are not significant from zero based on 95% confidence limits.

*Sp* Moho depths on the rift flanks in the northern segment generally agree with depths from *Ps* waveform fitting and *H‐κ* stacking (Zhang et al., 2016; Figure 8a). *Sp* Moho depths on the rift flanks in the middle segment sometimes agree and are also shallower than those from *Ps* (Zhang et al., 2016; Figure 8b). Beneath the rift axis in the northern segment, depths of the *Sp* Moho phase (36–40 ± 4 km) agree with the depths from the *H‐κ* stacking of Zhang et al. (2016). The *Sp*‐binned grid does not resolve a deeper (up to 60 km) discontinuity found using waveform fitting of the *Ps* receiver functions and interpreted as the base of crustal underplating (Zhang et al., 2016). Similarly, *Sp* results from the middle rift segment exhibit Moho phase depths of 35–37 ± 4 km in agreement with the *H‐κ* stacking depths but not the base of crustal underplating at up to 60‐km depths from waveform modeling (Zhang et al., 2016). However, *Sp* does image two distinct phases beneath the rift at 34–39 ± 4 and 62–65 ± 4 km in the southern segment similar to the interpreted *Ps* structure in the northern and middle segments, although the *Ps* study did not extend as far south as the location of the double *Sp* discontinuity.

The weaker *Sp* Moho amplitudes beneath the rift axis in the northern and middle segments (Figure 4 and cross sections A–A′ and C–C′, Figure 5) suggest a gradational seismic velocity increase with depth (Figures 3a and 3c, red and purple). This is generally consistent with the weaker and less coherent *Ps* phases reported for SPREE stations located on the rift (Zhang et al., 2016). We suggest that the single gradational velocity increase that we image here represents the boundary between prerift crust and deeper crustal underplating. Geophysical interpretations of an active‐source seismic profile also show an underplated layer down to 55 km beneath the Lake Superior portion of the rift (Cannon et al., 1989; Shay & Trehu, 1993). The Lake Superior portion of the rift is not included in this study; however, similar crustal features have been inferred along the southwestern arm of the MCR with gravity and magnetic surveys (Allen et al., 2006), and therefore, the two portions of the rift may be comparable. Magmatic underplating of the crust is able to explain a deeper discontinuity (such as that found by Zhang et al., 2016, at 55–60‐km depth and in our stack of *Sp* receiver functions from 55° to 60° epicentral distances) and is supported by high magmatic activity during rifting (Hutchinson et al., 1990; White, 1997). A possible cause for the general lack in our *Sp* results of a deeper phase at ~60 km in the northern and middle rift segments is that lower inherent frequencies of *S* waves set a limit on how close two discontinuities can be to be resolved as two phases (Rychert et al., 2005, 2007). However, synthetic tests show that two discontinuities placed ~30 km apart in depth, as may be expected here, produce two distinct phases (see Figures 3b and 3d, green and dark blue). More likely is that the structure of the crustal underplating is complex in these sections of the rift, possibly with strong lateral variations and steep dips, so that we do not observe a coherent phase that would define the base of crustal underplating when stacking all the data here. In particular, the steeply dipping edges of the transitional layer imaged and interpreted as a crustal underplate by Zhang et al. (2016; Figure 8) are not likely to be resolvable with *Sp* receiver functions, based upon modeling of wave propagation and *Sp* conversions through synthetic models of laterally varying velocity structure (Lekić & Fischer, 2017).

The two relatively strong positive phases beneath the rift in the southern segment suggest one distinct layer, delineated by relatively sharp discontinuities at its top and bottom, possibly describing a similar layer of crustal underplating beneath prerift crust. A possible higher volume of magma in the southern segment, as modeled with gravity data (Merino et al., 2013), may have manifested in a more laterally extensive underplated body than beneath the northern segment, allowing our *Sp* receiver functions to produce coherent phases that define the top and bottom of the layer of crustal underplating. Alternatively, variations in the character of the crustal underplating may be due to the different segments of the rift having formed in different geological terranes that could have affected the mobility of the melt through the crust. Topography of the boundary of the underplated layer could also have been affected by differential uplift dependent on the orientation of the compressional forces related to the Grenville Orogeny after the rifting event (Zhang et al., 2016).

### 5.2 Negative Velocity Discontinuities

Depths dominant in the southern segment (90–120 ± 7 km) are consistent with a midlithospheric discontinuity (MLD; Ford et al., 2010) within the North American continental lithosphere (Abt et al., 2010; Hansen et al., 2015; Rychert & Shearer, 2009; Selway et al., 2015). The cause of this sharp velocity decrease is not well understood. Proposed causes include the following: elastically accommodated grain boundary sliding (Karato et al., 2015); an anisotropic boundary between a highly depleted chemical lid and a less depleted thermal layer underneath (Yuan & Romanowicz, 2010); and metasomatism of lithospheric mantle rocks to produce seismically slower hydrous minerals, creating a frozen‐in layer of volatile‐rich melt (Hopper & Fischer, 2015; Selway et al., 2015).

Other *Sp* analyses in the region also finds a velocity decrease with depth at 70–110 km, interpreted as the MLD (Foster et al., 2014; Hansen et al., 2015), and a deeper phase at 200–240 km, interpreted as the lithosphere‐asthenosphere boundary (LAB; Foster et al., 2014). The depth range, 150–190 ± 7 km, predominant in the north half of our study region where we image a negative phase agrees with the lithospheric thickness estimates from Rayleigh waves and receiver functions for the Superior Province (Darbyshire et al., 2007) and also that from SS precursors (Tharimena et al., 2017). The deeper phases in our 150–190 ± 7‐km depth range may agree with the LAB of Foster et al. (2014), although these phases are few and the coverage is patchy (Figure 7). The *Sp* receiver function analysis of the US using Earthscope Transportable Array, SPREE, and other permanent stations also observes very few phases in this depth range (Hopper & Fischer, 2018). The negative phase that we observe at a depth of around 150 ± 7 km is much shallower than LAB depths observed in other *Sp* studies (Foster et al., 2014), determined by depth constraints of azimuthal anisotropy across the continent (Yuan & Romanowicz, 2010), and estimated by teleseismic *P* wave tomography with the MCR on the edge of the study (Frederiksen et al., 2013).

The depth variability of the negative velocity discontinuities illuminated by *Sp* receiver functions here does not correspond spatially to the MCR. Instead, it seems more related to the Spirit Lake Tectonic Zone, which separates the Yavapai Province (1.8–1.7 Ga) from the Minnesota River Valley Subprovince (3.6–3.4 Ga) and the Penokean Orogeny (1.9–1.8 Ga; Holm et al., 2007; Shen et al., 2013; Figure 7). Particularly in the center of the middle segment, the overlap of the two negative phases coincides with the Spirit Lake Tectonic Zone (cross section E–E′; Figure 6). The main negative phase at 90–120 ± 7‐km depth in the southern and middle segments is also north dipping. The dipping feature may be related to the relict subduction zone from the accretion of the Yavapai Province to the Superior Province ~1.7 Ga (Hopper & Fischer, 2015; Thurner et al., 2015). These are therefore likely features that predate the MCR and furthermore do not seem to exhibit rift‐related modification in what we image. Using Earthscope long period magnetotelluric data, Yang et al. (2015) also find no clear electrical resistivity anomalies associated with the MCR in the mantle. Lack of rift‐related alteration in our negative discontinuities may be explained by lithospheric healing or compression since the rifting event. Alternatively, rifting may have been accommodated by a narrow magmatic plumbing system that we are unable to resolve in our study.

### 5.3 Passive or Active Rifting?

Geochemical studies of well‐preserved outcropped igneous rocks related to the MCR demonstrate evidence of an enriched mantle source for volcanism during the rifting event (Davis & Green, 1997; Nicholson et al., 1997; Vervoort et al., 2007; White, 1997). This suggests an active rifting environment for the MCR, with a plume possibly having been centered beneath Lake Superior with a radius of up to 600 km (Allen et al., 1992). The present East African Rift is considered to have formed in an active rifting regime (Rychert et al., 2012) and has features that are similar to the MCR. As well as its arm structure, the East African Rift shows crustal thinning beneath its extending arms (Simiyu & Keller, 1997), similar to what the MCR is believed to have experienced during rifting prior to its crustal rethickening. Furthermore, in a passive rifting regime, greater degrees of lithospheric thinning are expected (Huismans & Beaumont, 2011), even over the short lifetime of the MCR of ~20 Myr (Vervoort et al., 2007). Instead, we image negative velocity discontinuities in the mantle lithosphere that likely predate the MCR with a lack of observed perturbations spatially related to the rift, particularly in the north‐dipping negative discontinuity (cross section E–E′; Figure 6) that is likely related to Yavapai accretion (Hopper & Fischer, 2015; Thurner et al., 2015). Additionally, our constraints on an altered Moho and addition of crustal underplating over a relatively focused, small lateral area beneath the rift axis supports focused magmatism at ~60‐km depth, which is more consistent with an active component of upwelling.

## 6 Conclusions

Here we use *S*‐to‐*P* receiver functions from SPREE, the Earthscope Transportable Array, and the US backbone network to constrain Moho and lithospheric discontinuity structure beneath the southwestern arm of the 1.1‐Ga MCR and its flanks. We illuminate a relatively flat positive seismic velocity increase with depth over the region at depths of 33–40 ± 4 km associated with the Moho, with little or no rift‐flank depth variation. Beneath the rift in the northern half of the study region, receiver function amplitudes are generally weak for the phase at Moho depths, indicating a more gradual velocity increase with depth, or a Moho with considerable relief. A deeper positive discontinuity at 61 ± 4 km beneath the rift in the north is present in a subset of the data, possibly because it is weaker, more steeply undulating, and/or not laterally coherent over a wide area. A second velocity increase at 62–65 ± 4 km also exists beneath the rift in the southern half of the study region, coupled with a generally sharper velocity discontinuity at Moho depths than in the north. The ability to resolve a layer of crustal underplating in the south suggests that it is more laterally extensive than in the north, which could be due to more magmatism during rifting in the south, or that more melt mobility was permitted in the north. We also image two velocity decreases with depth in the mantle lithosphere. In the southern half of the study region, a north‐dipping negative discontinuity exists at depths of 90–120 ± 7 km, which we attribute to old provincial suturing that predates the MCR and is consistent with depths of a MLD revealed in other studies. The northern half of the study region hosts a less spatially consistent negative discontinuity at a depth range of 150–190 ± 7 km that might be the LAB. We do not observe signs of lithospheric mantle alteration related to the MCR, suggesting that the lithosphere has since healed, been compressed post‐rift, or that we are unable to resolve potentially narrow signatures of past upwelling mantle material.

## Supporting information

Supporting Information S1

Data Set S1
