# Supplementary material for: Seismic Imaging of the North American Midcontinent Rift Using S‐to‐P Receiver Functions
Source: J Geophys Res Solid Earth. 2018 Sep 19;123(9):7791–805. doi: 10.1029/2018JB015771 (PMC6473666; doi:10.1029/2018JB015771)
Supplement: Supplementary file 1 — Supporting Information S1 [file JGRB-123-7791-s001.pdf]

Journal of Geophysical Research: Solid Earth

Supporting Information for

Seismic Imaging of the North American Mid-Continent Rift using S-to-P Receiver Functions

Ben Chichester[1], Catherine Rychert[1], Nicholas Harmon[1], Suzan van der Lee[2], Andrew Frederiksen[3], Hao Zhang[4]

[1] National Oceanography Centre Southampton, Ocean and Earth Sciences, University of Southampton, Southampton, UK.

[2] Department of Earth and Planetary Sciences, Northwestern University, Evanston, Illinois, USA.

[3] Department of Geological Sciences, University of Manitoba, Winnipeg, Manitoba, Canada.

[4] Department of Geology and Geophysics, University of Utah, Salt Lake City, Utah, USA.

Contents of this file

Figures S1 to S8

Additional Supporting Information (Files uploaded separately)

Caption for Data Set S1

Introduction

The supporting information contains eight supplementary figures and one dataset. Figures S1-S5 are hit-counts of cross-sections through the grid of stacked *S*-to-*P* (*Sp*) receiver functions related to the profiles A-A′ to E-E′ used in the main text. Figures S6-S8 show deeper receiver function results for cross-sections A-A′ to C-C′ that are not shown in the main text. Supplementary Data Set S1 is a Matlab MAT-file (.mat) containing the $0.25° \times 0.25° \times 1\ \mathrm{km}$ grid of migrated and stacked *Sp* receiver functions.

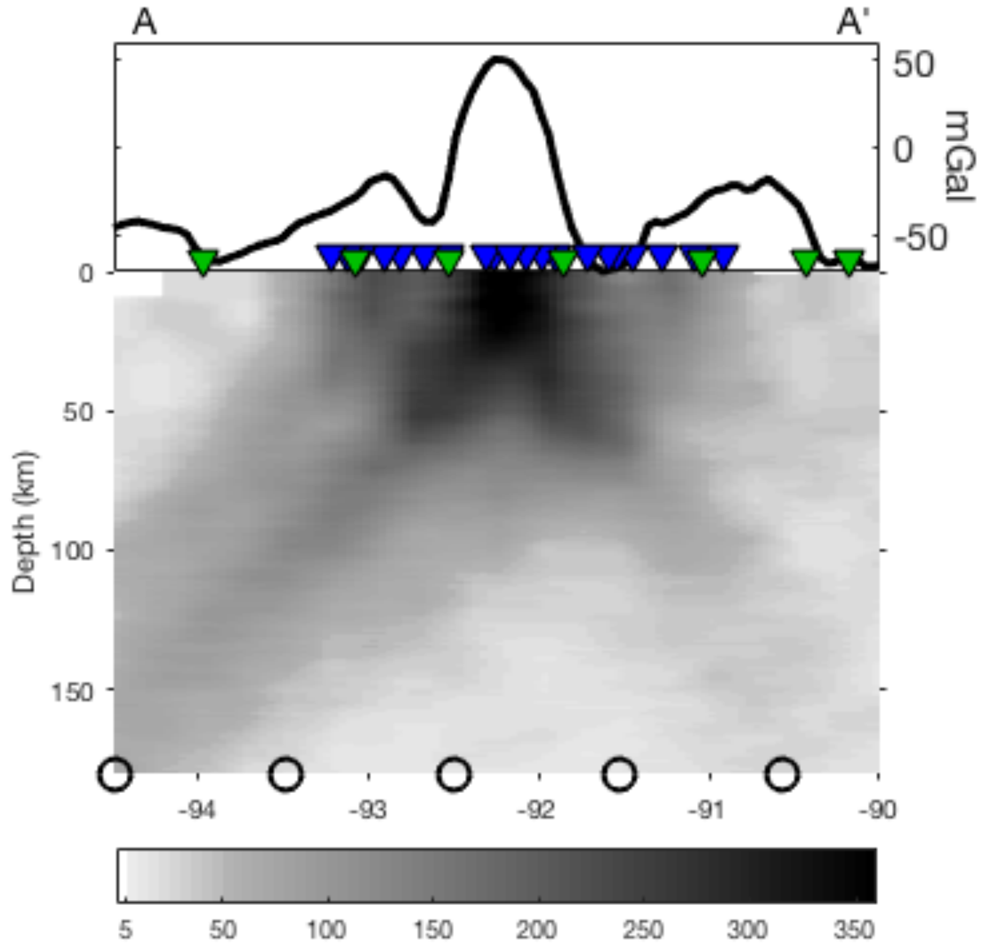

Figure S1. *Sp* receiver function hit count cross-section A-A', of which we show the migrated receiver function amplitude-depth profile in the main text. Grey scale shows number of receiver functions stacked into each bin in the profile cutting through the 3-D grid of stacked receiver functions. White regions in the main section of the plot indicate bins with <5 hits. Bouguer anomaly is plotted above each cross section in units of mGal [*Kucks,* 1999], where proximal stations are also indicated as inverted triangles – blue, SPREE; green, Earthscope Transportable Array and US backbone. Circles plotted at depth have 100 km lateral spacing and correspond to circles along the lines in main text Figures 1, 2 & 4.

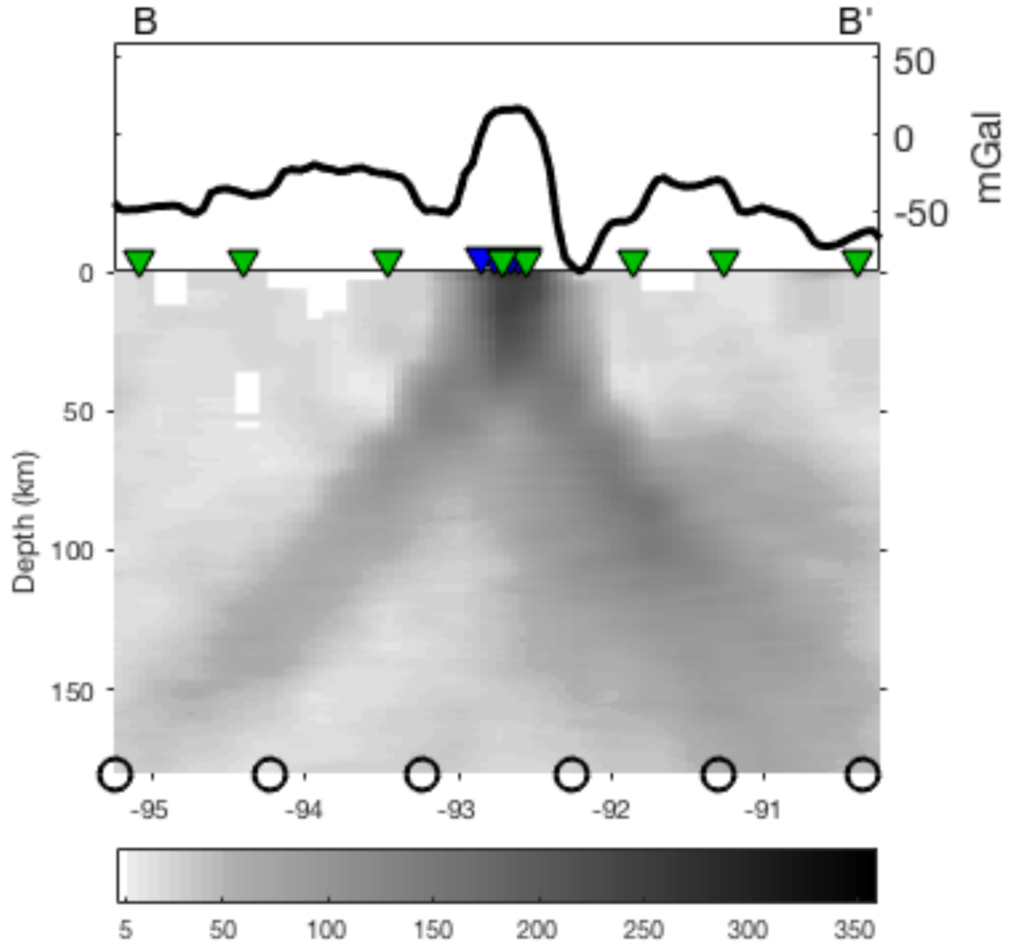

Figure S2. *Sp* receiver function hit count cross-section B-B′.

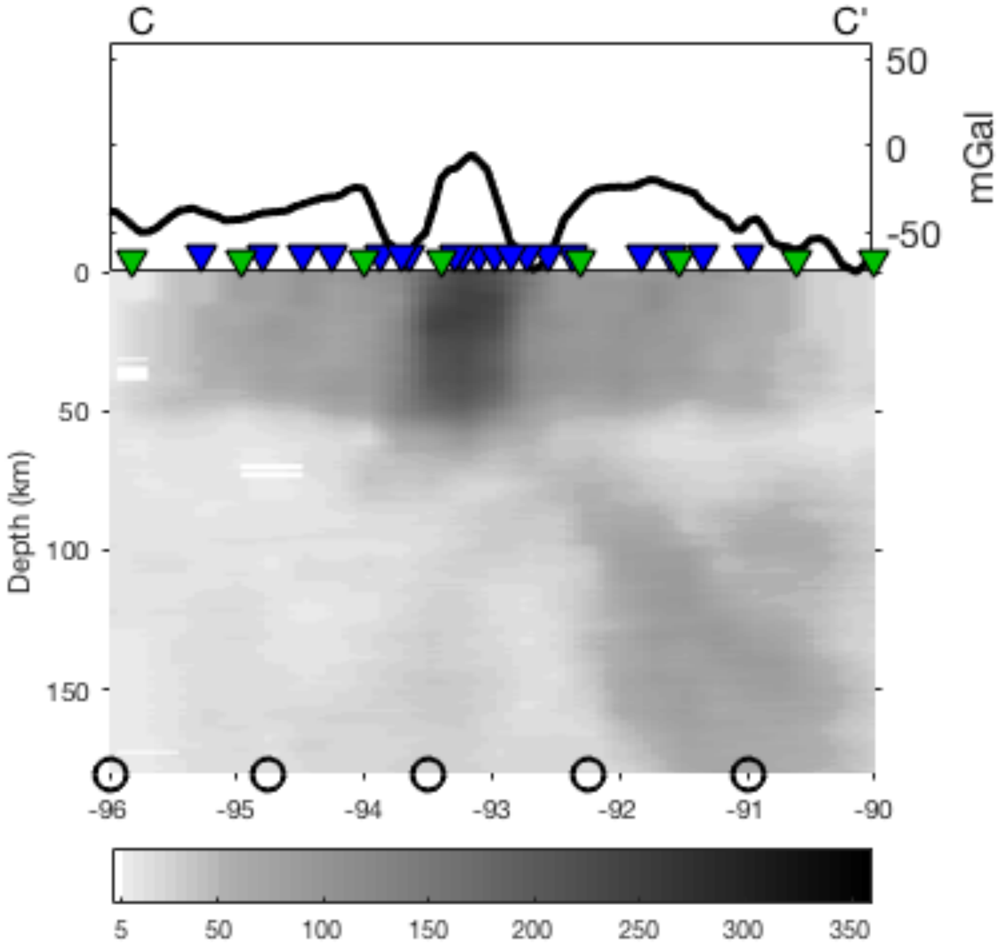

Figure S3. *Sp* receiver function hit count cross-section C-C′.

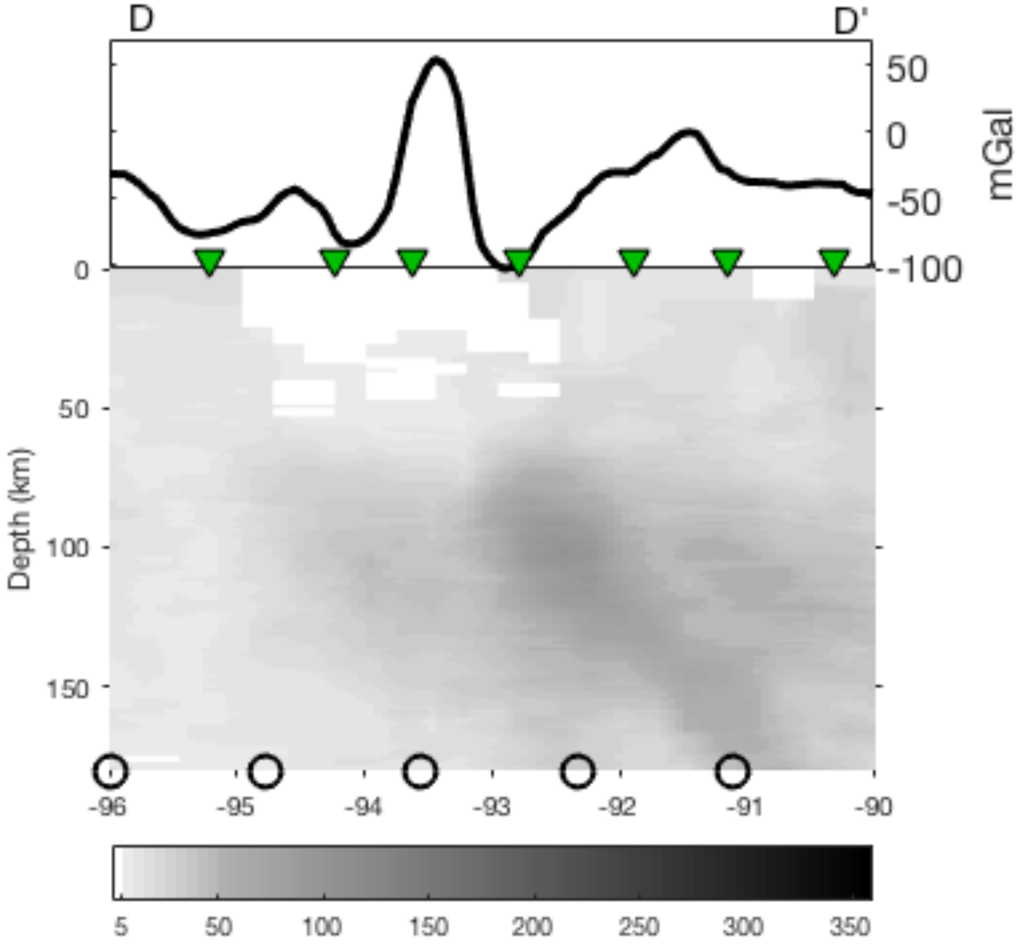

Figure S4. *Sp* receiver function hit count cross-section D-D′. Reference circles also shown on profile lines in Figure 7.

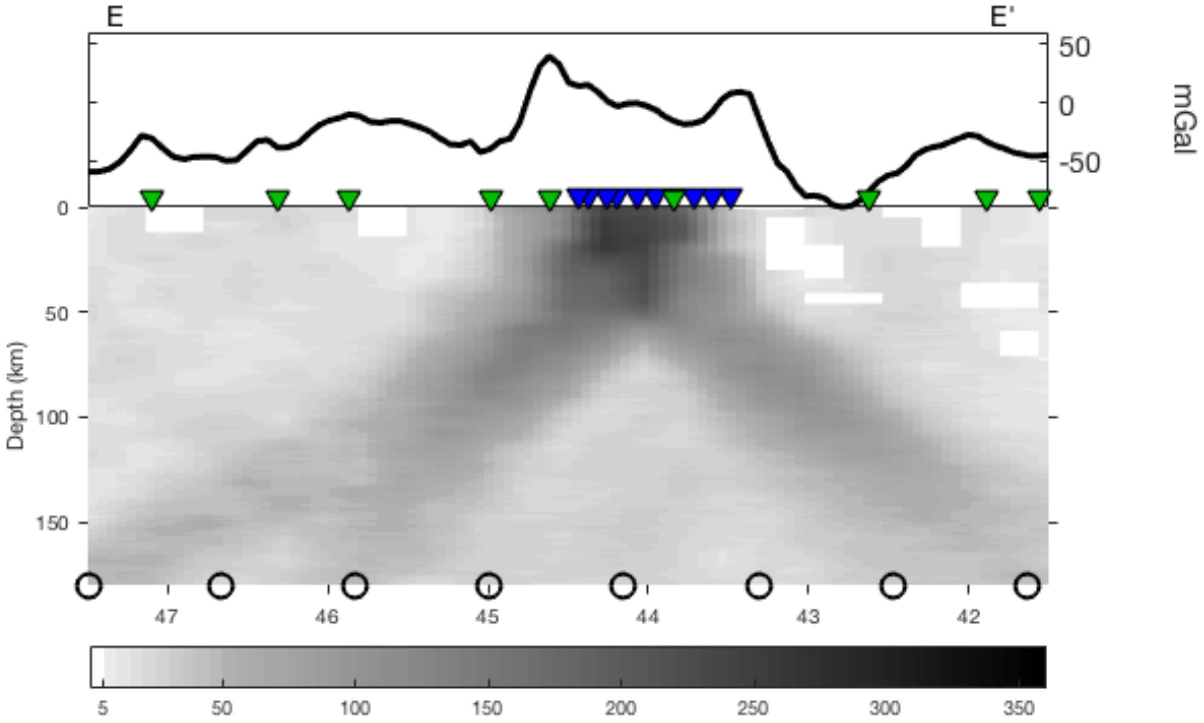

Figure S5. *Sp* receiver function hit count cross-section E-E′. Reference circles also shown on profile lines in Figure 7.

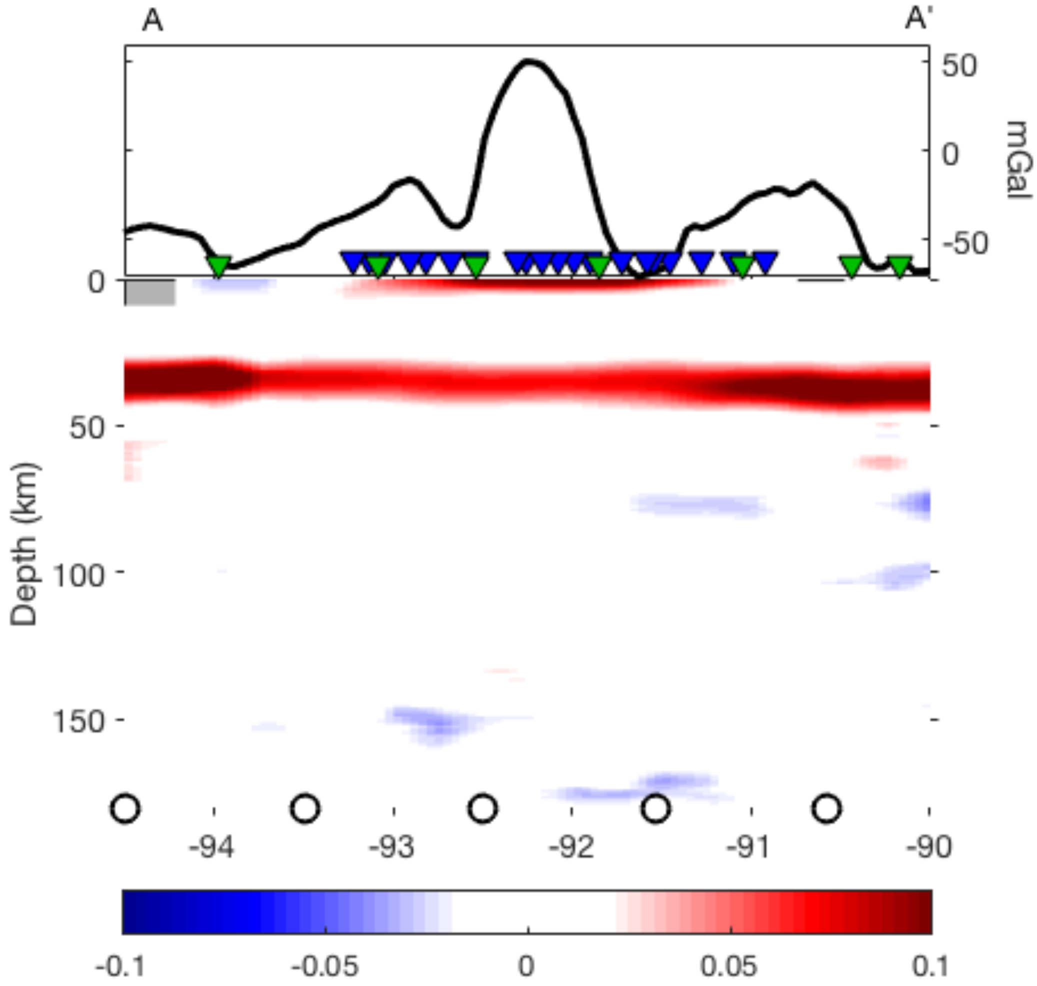

Figure S6. Cross-section A-A' showing migrated *Sp* receiver function phases at depth. The colours indicate the polarity of the seismic discontinuities from receiver functions: red, positive polarity (seismic velocity increase with depth); blue, negative polarity (seismic velocity decrease with depth). Amplitude colour bars are shown below each cross section. Circles plotted at depth have 100 km lateral spacing and correspond to circles along the lines in main text Figures 1, 2 & 4. Grey boxes signify bins that have <5 hits. Bouguer anomaly is plotted above each cross section in units of mGal [*Kucks,* 1999], where proximal stations are also indicated as inverted triangles – blue, SPREE; green, Earthscope Transportable Array and US backbone.

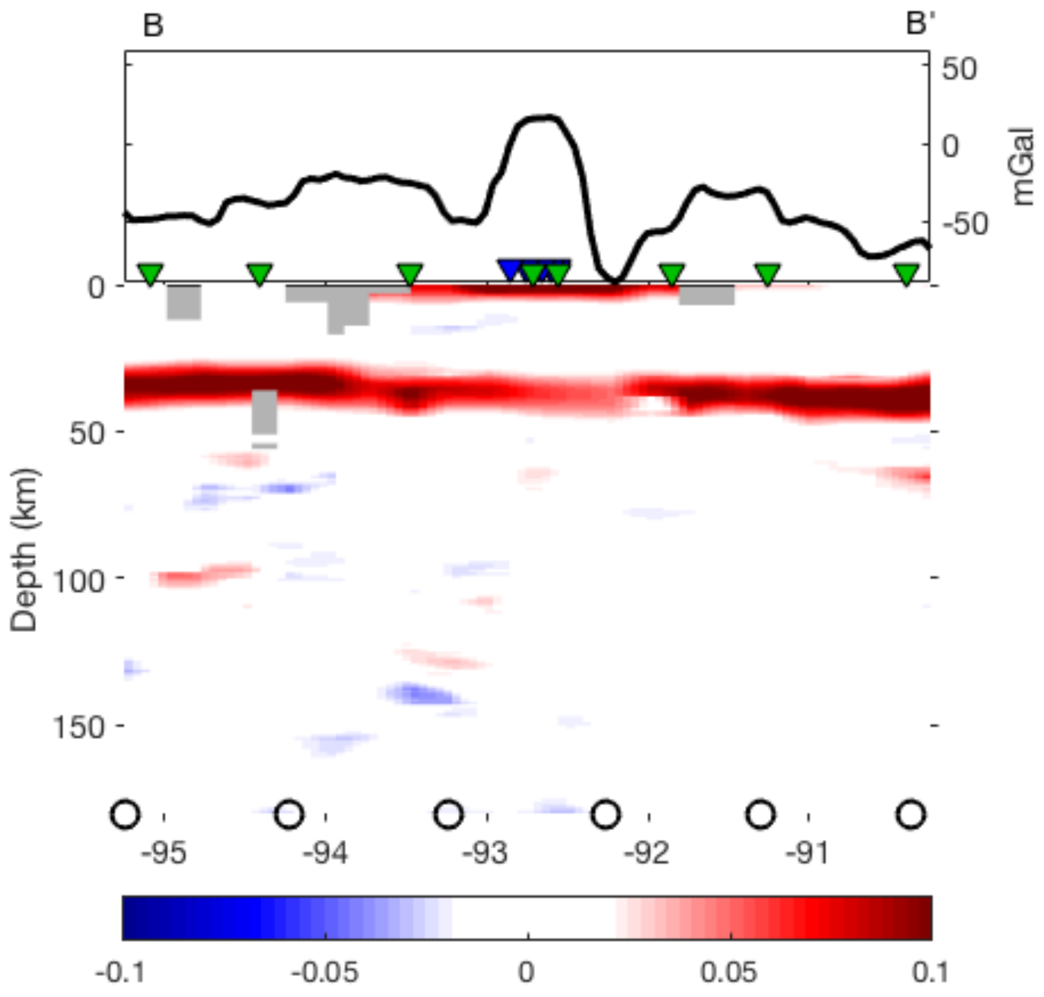

Figure S7. Cross-section B-B′ showing migrated *Sp* receiver function phases at depth.

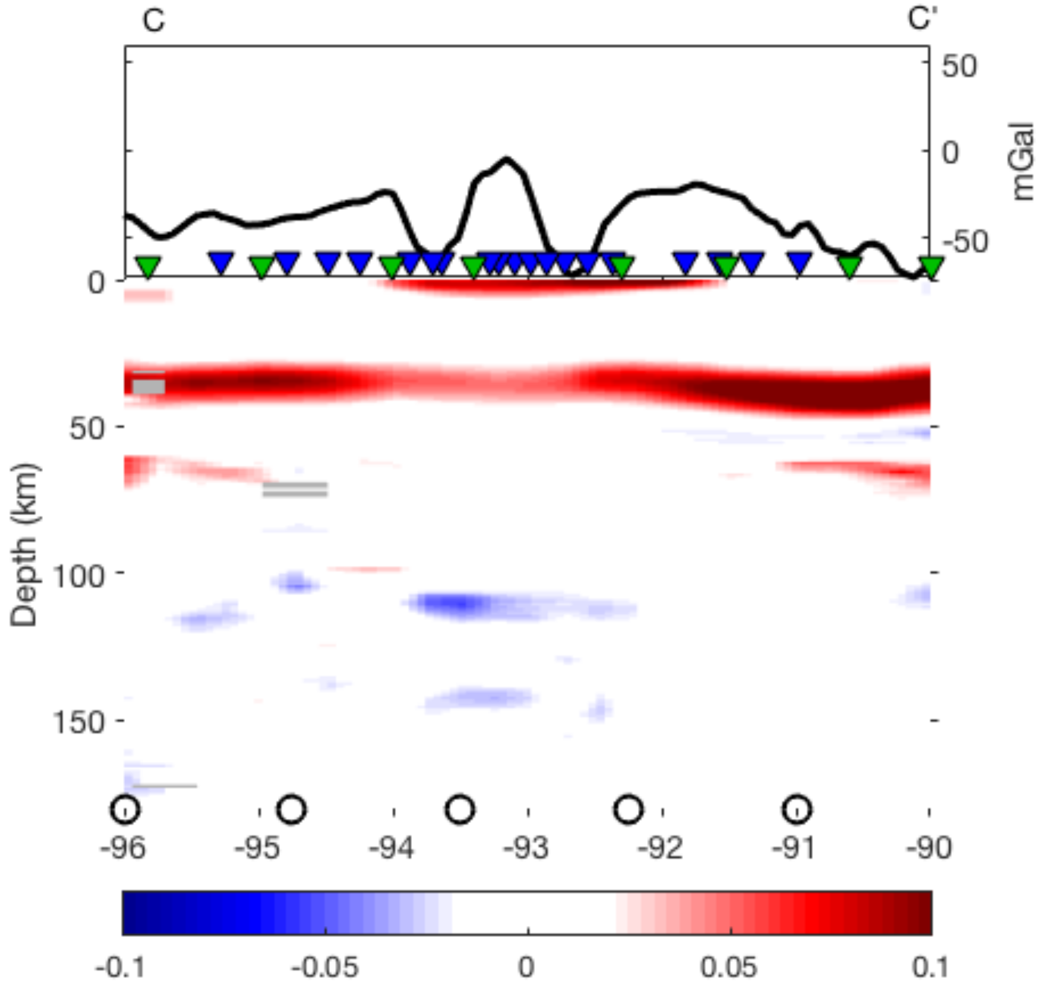

Figure S8. Cross-section C-C′ showing migrated *Sp* receiver function phases at depth.

Data Set S1. Matlab MAT-file of the 3-D volume of the migrated and stacked *Sp* receiver functions (ds01_SRF_3Dvol.mat). The grid size is $49 \times 93 \times 301$, representing a region over latitudes 40°-52° N and longitudes 100°-70° W both with 0.25° spacing, and with depths 0-300 km with 1 km spacing. All cross-sections and maps of the *Sp* receiver function phases in the main text and Figures S6-8 are created from this grid.
